# LCZ696 Protects against Diabetic Cardiomyopathy-Induced Myocardial Inflammation, ER Stress, and Apoptosis through Inhibiting AGEs/NF-κB and PERK/CHOP Signaling Pathways

**DOI:** 10.3390/ijms23031288

**Published:** 2022-01-24

**Authors:** Osamah M. Belali, Mohammed M. Ahmed, Mohamed Mohany, Tarig M. Belali, Meshal M. Alotaibi, Ali Al-Hoshani, Salim S. Al-Rejaie

**Affiliations:** 1 Department of Pharmacology and Toxicology, College of Pharmacy, King Saud University, Riyadh 11451, Saudi Arabia; osamabelali@yahoo.com (O.M.B.); mmahmed114@yahoo.com (M.M.A.); mmohany@ksu.edu.sa (M.M.); ahoshani@ksu.edu.sa (A.A.-H.); 2 Faculty of Applied Medical Sciences, University of Bisha, Bisha 67714, Saudi Arabia; blaly@ub.edu.sa; 3 Department of Radiological Technology, College of Applied Medical Sciences, King Saud University, Riyadh 11451, Saudi Arabia; mbakheet@ksu.edu.sa

**Keywords:** LCZ696, valsartan, diabetic cardiomyopathy, advanced glycation end products, apoptosis, endoplasmic reticulum stress

## Abstract

The present study is designed to determine the effect of LCZ696 on DCM in rats and investigate the underlying mechanism involved. Diabetes was induced by feeding rats with a high-fat diet for six weeks following a single injection of STZ (30 mg/kg). Diabetic rats were divided into three groups (*n* = 10). LCZ696 and valsartan treatment was started two weeks after diabetic induction and continued for eight weeks. At the end of the treatment, serum and cardiac tissues were analyzed by RT-PCR, Western blot, and ELISA kits. LCZ696 and valsartan ameliorated DCM progression by inhibiting AGEs formation at activity levels; pro-apoptotic markers (BAX/Bcl2 ratio and caspase-3) in mRNA and protein expressions, the NF-κB at mRNA; and protein levels associated with the restoration of elevated proinflammatory cytokines such as the TNF-α, IL-6, and IL-1β at the activity level. Furthermore, LCZ696 and valsartan contribute to restoring the induction of ER stress parameters (GRP78, PERK, eIF2a, ATF4, and CHOP) at mRNA and protein levels. LCZ696 and valsartan attenuated DCM by inhibiting the myocardial inflammation, ER stress, and apoptosis through AGEs/NF-κB and PERK/CHOP signaling cascades. Collectively, the present results reveal that LCZ696 had a more protective solid effect against DCM than valsartan.

## 1. Introduction

Diabetes mellitus (DM) is one of the fastest-growing global health problems, becoming an epidemic in several countries, particularly Saudi Arabia. According to the International Diabetes Federation, 3,852,000 people developed diabetes in 2017 in Saudi Arabia, making it the seventh highest rate of diabetes in the world [1]. Diabetes is associated with several complications, including cardiovascular problems [2]. Several reports have shown that cardiovascular complications are the primary cause of death among diabetic patients [3,4]. In 1972, Rubler et al. described heart failure (HF) in postmortem diabetic patients as a disease that occurred in the absence of a coronary artery or valve disease called diabetic cardiomyopathy (DCM) [5]. The pathogenesis of DCM is complex and comprises a wide range of features, including a disruption of insulin secretion, glucose, and lipid metabolic process, as well as inflammatory responses [6]. Clinical manifestations of DCM in diabetic subjects include metabolic disorders, cardiac cell abnormalities, and further myocardial cell apoptosis and fibrosis, leading to ventricular wall stiffness and cardiac dysfunction, resulting in heart failure and death in severe illness cases [7,8]. Furthermore, in the diabetic heart, cytokines and chemokines secreted by infiltrative proinflammatory macrophages and lymphocytes contribute to the development of cardiomyocyte hypertrophy and progressive fibrotic response, resulting in extracellular matrix accumulation and fibrosis [9].

Diabetes induces mitochondrial dysfunction and endoplasmic reticulum (ER) stress in cardiac tissues, leading to the activation of pro-apoptotic pathways [10]. In addition, DM initiates oxidative stress resulting in the induction of advanced glycation end-products (AGEs) and inflammatory progression, which promotes cardiac fibrosis and apoptosis [10]. AGEs are proteins or lipids that become glycated because of their exposure to sugars, altering their functional properties. Persistent hyperglycemia and hyperinsulinemia promote AGEs formation, which enhances structural and functional alterations, leading to increased myocardial stiffness [11]. AGEs can also cause crosslinks in cardiac collagen, which leads to fibrosis [12]. In this regard, hyperglycemia induces AGEs formation and reactive oxygen species (ROS), which initiate proinflammatory responses via the nuclear factor kappa-light-chain-enhancer of activated B cells (NF-κB). AGEs bind to their receptor (RAGE) and directly activate the NF-κB [11]. In response to hyperglycemia, ROS are released into the ER, leading to ER stress by activating three independent ER stress sensors: activating transcription factor 6 (ATF6), inositol-requiring enzyme (IER1), and protein kinase R (PKR)-like endoplasmic reticulum kinase (PERK). These pathways induce the pro-apoptotic cascade and lead to cardiac hypertrophy and DCM. PERK activation leads to the auto-phosphorylation of downstream signaling molecules such as activating transcription factor 4 (ATF4) and eukaryotic initiation factor 2 (eIF2), thus activating the transcription of C/EBP homologous protein (CHOP) [13]. In a rat model of post-myocardial infarction, CHOP was considerably increased, and apoptosis was also detected in myocardial cells [14]. In addition, atorvastatin has been reported to attenuate myocardial cell apoptosis in rats by down-regulating the ER stress response and reducing caspase 12 and CHOP expression [14].

LCZ696 is a combination of valsartan and AHU377 (sacubitril) in a ratio of 1:1, which is synthesized by a complex chemical reaction. It inhibits the excessive activation of the renin–angiotensin–aldosterone system (RAAS) while simultaneously increasing the cardiovascular protection provided by the natriuretic peptide system. LCZ696 is a first-in-class angiotensin receptor neprilysin inhibitor approved by the FDA in 2015. The National Institute for Health and Care Excellence (NICE) guidance recommended LCZ696 for treating symptomatic chronic HF with reduced ejection fraction, i.e., left ventricular ejection fraction of 35% or less. Clinical studies have reported that LCZ696 is superior to enalapril in moderate to severe HF [15,16]. Therefore, we hypothesized that LCZ696 treatment would demonstrate beneficial effects against DCM in the diabetic type-2 rat model by reducing myocardial inflammation, ER stress, and apoptosis. We proposed to investigate the role of the AGEs/NF-κB and PERK/CHOP pathways in the LCZ696-mediated effects.

## 2. Results

### 2.1. Effect of LCZ696 and Valsartan Treatments on Weekly Body Growth and Heart Weight

**Figure 1:**
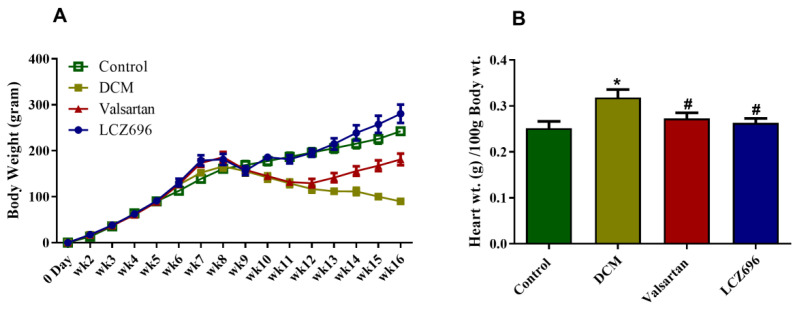
Effect of LCZ696 and valsartan treatments on weekly body weight and heart weight of control and diabetic rats. Data are expressed as mean ± SEM (*n* = 6) and analyzed using one-way ANOVA followed by Student–Newman–Keuls as a post hoc test. * DMC vs. control, # LCZ696 and valsartan vs. DMC. (**A**) weekly delta body weight, (**B**) heart weight (g) per 100 g body weight.

Experiments were established using rats weighing 100–120 g. In the seventh week, all the animal groups (DCM, LCZ696, and valsartan) exhibited a significant increase in the delta body weight compared to the control group. In week 10, the DCM and valsartan group exhibited a marked reduction in the delta body weight compared to the control, while the LCZ696-treated animal resisted that reduction. Compared to the DCM animal group, the delta body weight increased significantly in the LCZ696 and valsartan groups, starting from week 13 and week 10, respectively. The LCZ696-treated animals showed more body weight gain starting at week ten than the valsartan treated rats (Figure 1A).

At the end of the experiment, the heart was immediately dissected and weighed for each animal. The heart weight (HW; g) was calculated per 100 g of body. The results show that HW/100 g BW markedly increased, reaching approximately 21% compared to the untreated control. LCZ696 and valsartan treatments reduced the increase in diabetic rats. That suggests the possibility that LCZ696 results in restoring the HW/100 g BW in DCM rats. However, both drugs impacted the HW equally without significant variation compared to each other (Figure 1B).

### 2.2. Effect of LCZ696 and Valsartan Treatment on Blood Pressure and Cardiac Function in Diabetic Cardiomyopathy Rats

SBP and DBP levels were measured by the tail-cuff technique at day 0, after 8 weeks of HFD feedings, and at week 16, the end of treatment. After 8 weeks, the SBP and DBP increased significantly (*p* < 0.001) in all HFD-fed animals compared to the control rats. At the end of treatment, SBP and DBP were significantly increased compared to the control animals. The valsartan and LZC696 treatments reduced the elevation of SBP and DBP significantly compared to DCM rats (Table 1).

**Figure 2:**
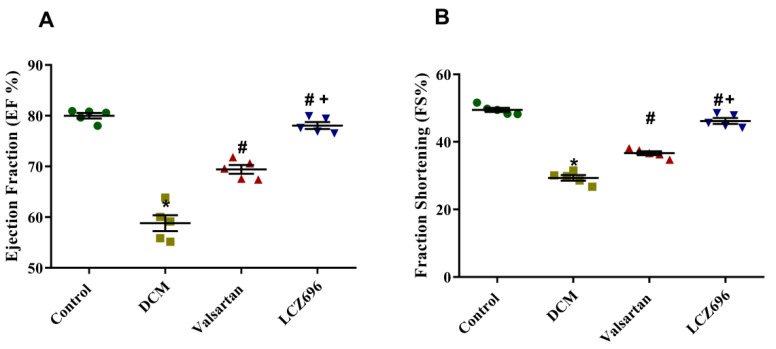
Effect of LZC696 on cardiac function in diabetic cardiomyopathy rat models. Echocardiography was used to examine (**A**) the ejection fraction (EF) and (**B**) fraction shortening (FS) of all experimental rats before the sacrificing day. Data are expressed as mean ± SEM (*n* = 5). Statistical analysis was conducted by one-way ANOVA followed by Student–Newman–Keuls as a post hoc test. The levels of significance were determined as: + *p* < 0.001, * *p* < 0.001, and # *p* < 0.001. * DCM vs. Control, # LCZ696 and valsartan vs. DCM, + LCZ696 vs. valsartan.

Echocardiography was used to examine the EF and FS of all experimental rats before the sacrificing day. As shown in Figure 2, the rats with HFD-STZ-induced diabetes developed ventricle dysfunction. The EF% and FS% of the DCM rats were significantly reduced compared with the control rats, while the EF% and FS% after the LCZ696 and valsartan treatments were higher than those of the DCM animals. The LCZ696 treatment was superior to valsartan in increasing EF% and FS% in diabetic animals (Figure 2).

### 2.3. Effects of LCZ696 and Valsartan on Serum Glucose, Insulin, and Cardiac Enzymes

**Figure 3:**
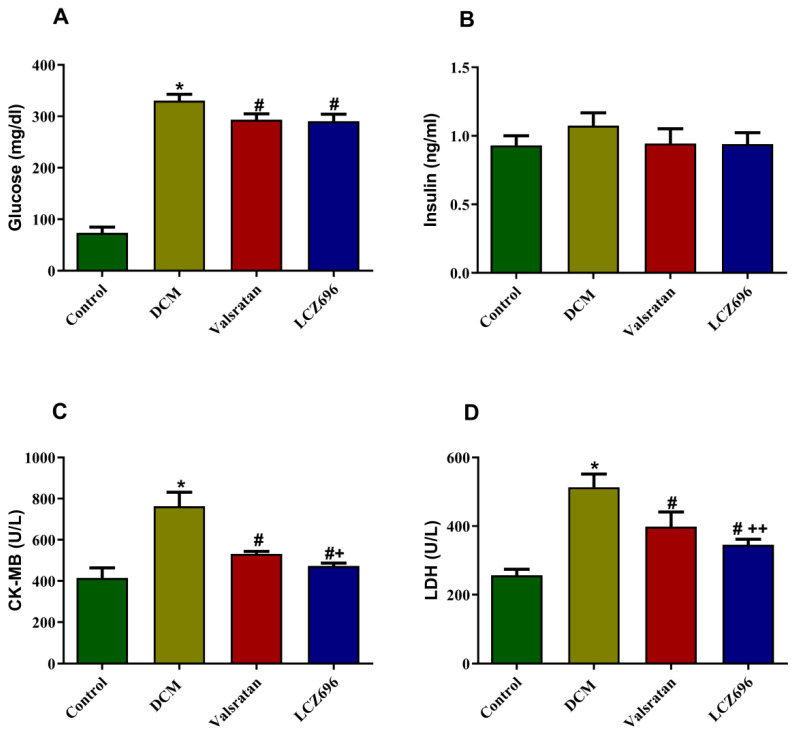
Effect of LCZ696 and valsartan on serum glucose (**A**), insulin (**B**), CK-MB (**C**), and LDH levels (**D**). Data are expressed as mean ± SEM (*n* = 6). Statistical analysis was conducted by one-way ANOVA followed by Student–Newman–Keuls as a post hoc test. The levels of significance were determined as: * *p* < 0.001, # *p* < 0.001, and ++ *p* < 0.01. * DCM vs. control, # LCZ696 and valsartan vs. DCM, + LCZ696 vs. valsartan.

Serum glucose levels were significantly elevated in the DCM group (*p* < 0.001) compared to the control group, and these values were significantly reduced in diabetic rats treated with LCZ696 (*p* < 0.001) and valsartan (*p* < 0.001) compared to the DCM rats. Plasma insulin levels did not demonstrate any substantial difference when compared to different groups. In order to investigate the hyperglycemia-induced cardiomyopathy and the potential effect of LCZ696 treatment, we measured the serum level of cardiac enzymes, including LDH and CK-MB. Circulation of LDH and CK-MB was markedly induced (*p* < 0.001) in the DCM animals compared to the control rats. The LCZ696 and valsartan treatments had the potential to significantly reduce this induction (*p* < 0.001). The effect of LCZ696 had a more pronounced and significantly elevated effect (*p* < 0.01 in CK-MB and *p* < 0.05 in LDH) compared to that in the valsartan-treated animals (Figure 3).

### 2.4. Effect of LCZ696 and Valsartan Treatments on Serum Lipid Profile 

**Figure 4:**
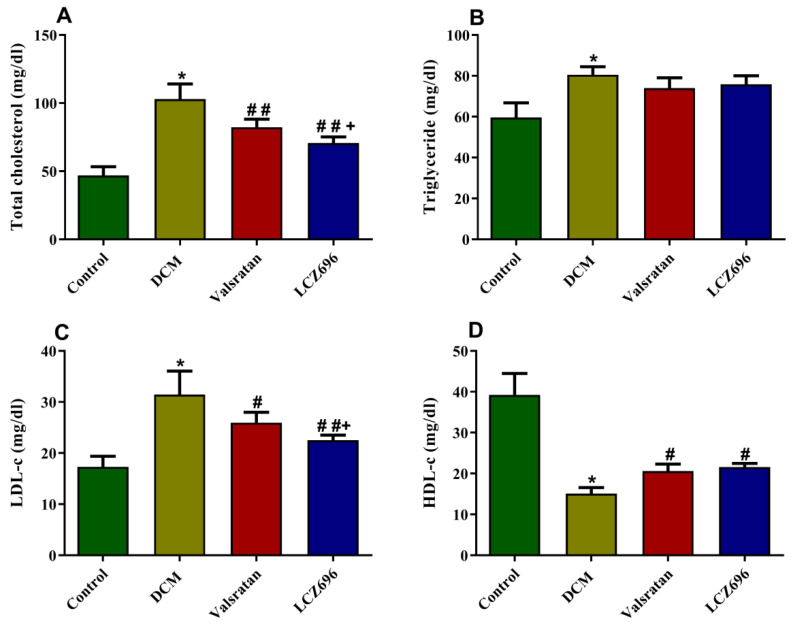
Effect of LCZ696 and valsartan on serum lipid profile. Data are expressed as mean ± SEM (*n* = 6). Statistical analysis was conducted by one-way ANOVA followed by Student–Newman–Keuls as post hoc test. The levels of significance in total cholesterol and triglyceride were determined as: * *p* < 0.001, ## *p* < 0.001, and + *p* < 0.05. In LDL-c and HDL-C: * *p* < 0.001, # *p* < 0.01, ## *p* < 0.001, and + *p* < 0.05. * DCM vs. Control, # LCZ696 and valsartan vs. DCM, + LCZ696 vs. valsartan.

The effect of LCZ696 and valsartan on serum lipids levels is displayed in Figure 4. With respect to the DCM group, the animals exhibited a marked (*p* < 0.001) induction in TC, TG, and LDL-c levels with a significant reduction in HDL-c. The LCZ696 and valsartan treatments significantly reduced the total cholesterol, LDL-c, and HDL-c (*p* < 0.01) without a substantial effect on hypertriglyceridemia. However, the LCZ696 treatment was superior to valsartan in reducing TC and LDL-c serum levels. 

### 2.5. Effect of LCZ696 and Valsartan Treatment on Inflammatory Biomarkers

**Figure 5:**
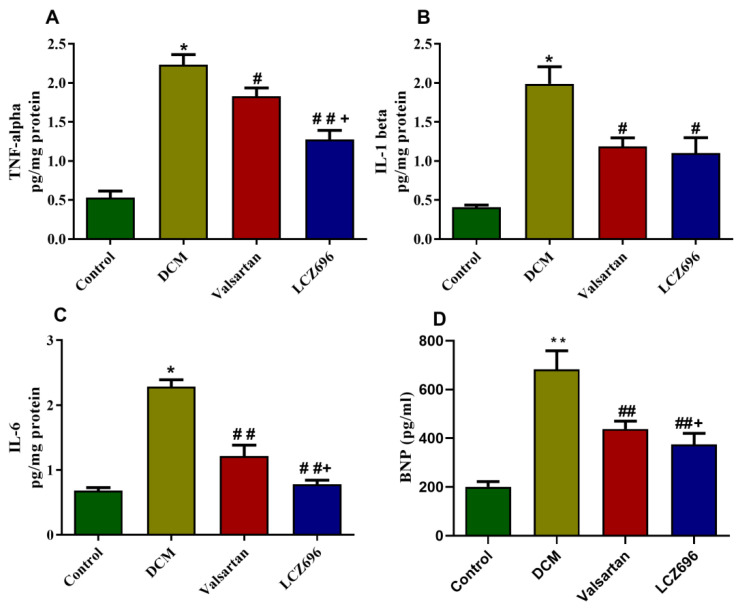
Effect of LCZ696 and valsartan on (**A**) tumor necrosis factor (TNF-α), (**B**) interleukin 6 (IL-6), (**C**) interleukin 1β (IL-1β) levels in cardiac tissues, and (**D**) serum BNP levels. Data are expressed as mean ± SEM (*n* = 6). Statistical analysis was conducted by one-way ANOVA followed by Student–Newman–Keuls as a post hoc test. The levels of significance were determined as: * *p* < 0.001, # *p* < 0.05, ## *p* < 0.001, + *p* < 0.05. * DCM vs. Control, # LCZ696 and valsartan vs. DCM, + LCZ696 vs. valsartan.

In cardiac tissue homogenates, proinflammatory cytokines levels were quantified and revealed a significant increase in the DCM group in the levels of the TNF-α, IL-6, and IL-1β (*p* < 0.001) compared to control rats. The LCZ696 and valsartan treatment of diabetic rats markedly reduced these cytokines levels compared to the DCM group. However, the LCZ696 treatment showed a more significant reduction (*p* < 0.05) in TNF-α and IL-6 levels compared to the valsartan-treated animals. Similarly, the serum levels of BNP were significantly increased (*p* < 0.01) in the DCM group compared to the control rats. This increase in the serum level of BNP in diabetic rats was also reduced by the administration of LCZ696 and valsartan, but the LCZ696-treated group exhibited a more significant reduction (*p* < 0.05) than the valsartan-treated group (Figure 5). These findings show that LCZ696 had anti-inflammatory and anti-heart failure properties in a DCM experimental paradigm.

### 2.6. Effect of LCZ696 and Valsartan Treatments on the mRNA and Protein Expressions of Apoptotic Genes including BAX, Bcl2, and Caspase-3 in Cardiac Tissues

**Figure 6:**
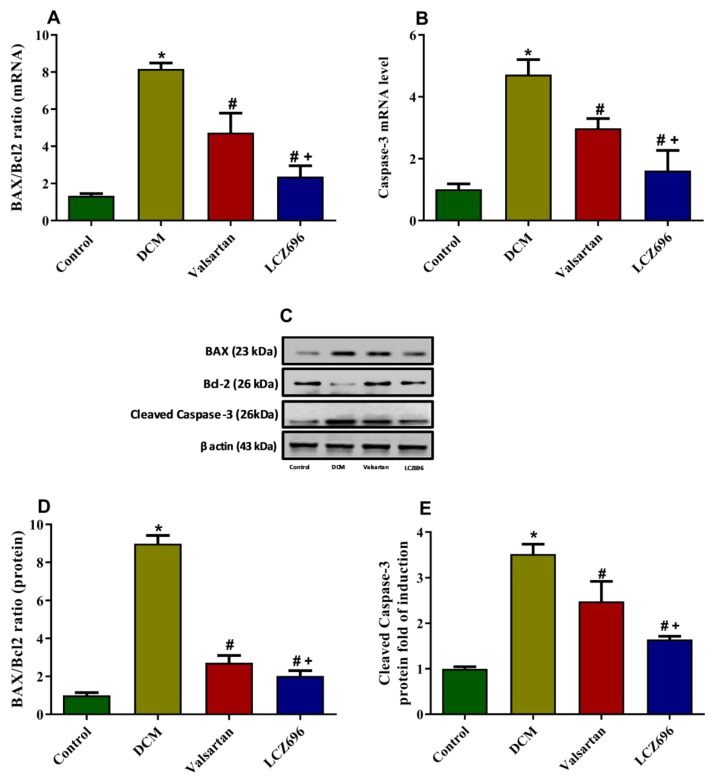
Effect of LCZ696 and valsartan on apoptotic genes mRNA (**A**,**B**) and protein expressions (**C**–**D**) in cardiac tissues. Data are expressed as mean ± SEM (*n* = 6 in mRNA and *n* = 3 in protein). Statistical analysis was performed by one-way ANOVA followed by Student–Newman–Keuls as a post-hoc test. The levels of significance were determined as: + *p* < 0.05, * *p* < 0.001, and # *p* < 0.001. * DCM vs. Control, # LCZ696 and valsartan vs. DCM, + LCZ696 vs. valsartan.

The BAX/Bcl2 ratio at mRNA and protein levels were significantly increased (*p* < 0.05) in the DCM group compared to the control group. The LCZ696 and valsartan treatment of diabetic rats markedly inhibited (*p* < 0.05) the BAX/Bcl2 ratio compared to that in the DCM animals. The effect of LCZ696 demonstrated a more protective effect against the DCM-induced inhibition of the BAX/Bcl2 ratio (*p* < 0.05) than the valsartan treatment (Figure 6A,C,D). The caspase-3 mRNA and protein levels were significantly increased (*p* < 0.05) in the DCM rats compared to the control group. The LCZ696 and valsartan treatment of DCM rats markedly (*p* < 0.05) inhibited the levels of caspase-3 mRNA in cardiac cells as compared to untreated animals. In the LCZ696-treated group, the caspase-3 mRNA and protein levels were significantly (*p* < 0.05) decreased compared to the valsartan-treated animals (Figure 6B,E).

### 2.7. Effect of LCZ696 and Valsartan on AGEs Accumulation and RAGE Expression Levels

**Figure 7:**
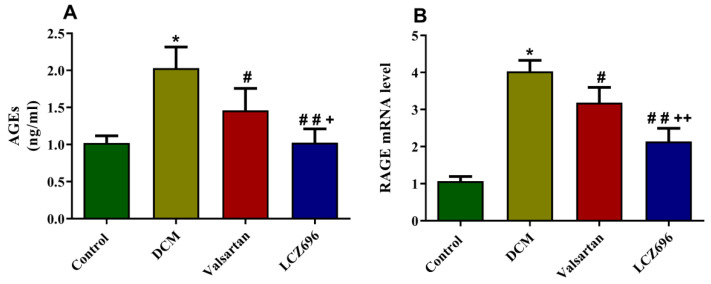
Effect of LCZ696 and valsartan on AGEs accumulation (**A**) and RAGE expressions (**B**) in cardiac tissues. The data are expressed as mean ± SEM (*n* = 6). Statistical analysis was conducted by one-way ANOVA followed by Student–Newman–Keuls as a post-hoc test. The levels of significance were determined as: * *p* < 0.001, # *p* < 0.05, ## *p* < 0.001, + *p* < 0.05, and ++ *p* < 0.01. * DCM vs. Control, # LCZ696 and valsartan vs. DCM, + LCZ696 vs. valsartan.

AGEs accumulation is one of the parameters known in diabetes that mediates the undesirable effect of hyperglycemia. To determine the ability of LCZ696 and valsartan to inhibit AGEs accumulation and RAGE expression, we quantified the amount of AGEs formation using a commercially available ELISA kit (Figure 7A) and RAGE mRNA using the RT-PCR method (Figure 7B) in cardiac tissues. The mRNA level of RAGE was significantly induced (*p* < 0.001) in the DCM rats compared to the controls. The LCZ696 and valsartan treatments of diabetic rats substantially decreased RAGE mRNA levels (*p* < 0.05 and *p* < 0.001, respectively) in cardiac cells compared to the DCM animals. In the LCZ696-treated group, the RAGE mRNA levels were significantly decreased (*p* < 0.05) compared to valsartan-treated animals. The AGEs accumulation levels were significantly increased (*p* < 0.001) in the DCM rats compared to the control group. The LCZ696 and valsartan treatments significantly inhibited the AGEs levels in cardiac cells compared to the DCM group of animals (*p* < 0.001 and *p* < 0.05, respectively). The LCZ696 treatment on DCM rats showed a higher (*p* < 0.01) protective effect against the accumulated AGEs than valsartan.

### 2.8. Effect of LCZ696 and Valsartan on NF-κB mRNA and Protein Expressions in Cardiac Tissues

**Figure 8:**
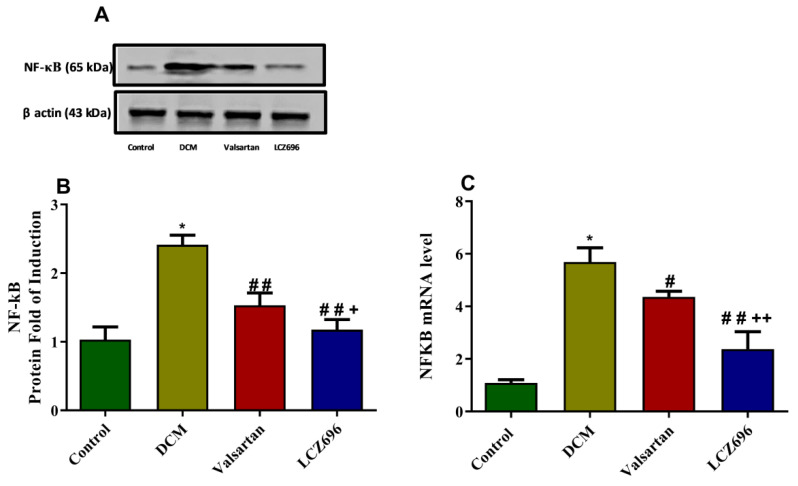
Effect of LCZ696 and valsartan on NF-κB mRNA and protein expressions in cardiac tissues. Data are expressed as mean ± SEM (*n* = 6 in mRNA and *n* = 3 in protein). Statistical analysis was conducted by one-way ANOVA followed by Student–Newman–Keuls as a post-hoc test. The levels of significance were determined as: * *p* < 0.001, # *p* < 0.01, ## *p* < 0.001, + *p* < 0.01, and ++ *p* < 0.01. * DCM vs. Control, # LCZ696 and valsartan vs. DCM, + LCZ696 vs. valsartan.

In order to identify the mechanism underlying the reduced AGEs formation and RAGE mRNA level, we investigated the mRNA and protein levels of the NF-κB in cardiomyocytes using RT-PCR and Western blot systems, respectively. NF-κB mRNA levels were increased in the DCM rats compared to the normal rats by approximately fivefold. The LCZ696 and valsartan treatment of DCM rats markedly inhibited the levels of NF-κB mRNA in cardiac tissue (*p* < 0.001 and *p* < 0.05, respectively) compared to the untreated DCM animals. In the LCZ696-treated group, the NF-κB mRNA levels significantly decreased (*p* < 0.01) compared to the valsartan-treated animals. Similarly, Western blot data revealed that NF-κB protein levels were increased significantly (*p* < 0.05) in the DCM rats compared to the control rats. LCZ696 and valsartan were able to significantly reduce this elevation (*p* < 0.001) in comparison with the DCM rats. The LCZ696 treatment had a greater significant decrease (*p* < 0.05) in NF-κB protein levels in cardiac tissue than diabetic animals treated with valsartan, as shown in Figure 8.

### 2.9. Effect of LCZ696 and Valsartan on Expressions of Endoplasmic Reticulum Stress Parameters

**Figure 9:**
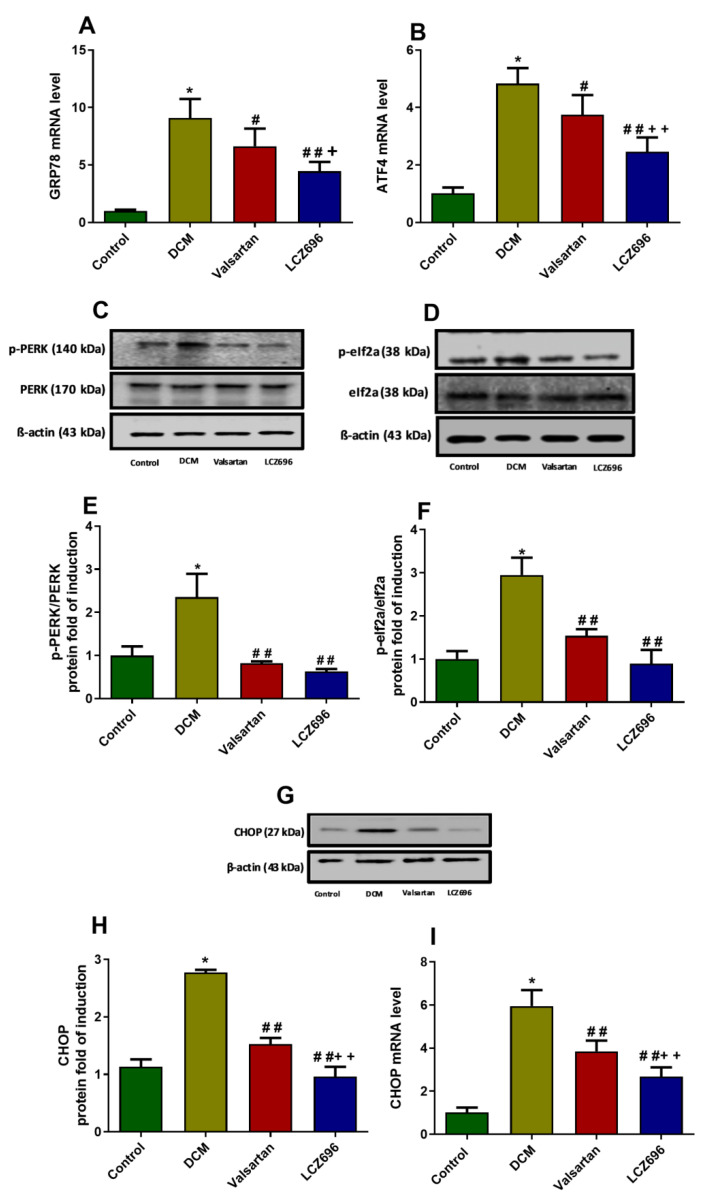
Effect of LCZ696 and valsartan on ER stress parameters in DCM rats. (**A**,**B**) The mRNA level of the GRP78 and ATF4 genes. (**C**–**F**) The protein expression level of *p*-PERK and *p*-eIf2a normalized to their total form. (**G**,**H**) CHOP protein expression normalized to β-actin. CHOP (**I**) RT-PCR results were normalized to β-actin housekeeping gene and data are expressed as mean ± SEM (*n* = 6). The protein expression level of ER stress parameters quantified by Western blot analysis and data are expressed as mean ± SEM (*n* = 3). The levels of significance were determined as: * *p* < 0.001, # *p* < 0.01, ## *p* < 0.01, + *p* < 0.05, and ++ *p* < 0.01. * Control group compared to DCM, # LCZ696 and valsartan compared to DCM, + LCZ696 compared to valsartan.

ER stress parameters were estimated and presented in Figure 9. The molecular chaperone GRP78 has an essential role in cardiac cells’ survival and development since its overexpression and accumulation have been considered as an indicator that reflects the initiation of ER stress. The mRNA expression levels of GRP78 expression levels were measured using RT-PCR. The mRNA levels of GRP78 were significantly induced (*p* < 0.001) in the DCM animal group compared to the control group. The LCZ696 and valsartan treatment of DCM rats significantly reduced the GRP78 mRNA levels compared to the untreated DCM animals. However, the LCZ696 treatment markedly (*p* < 0.05) reduced the elevated GRP78 mRNA levels compared to the valsartan treated animals (*p* < 0.001 and *p* < 0.01, respectively). PERK is an ER stress activation sensor that can also be activated by GRP78, leading to the phosphorylate of eIf2a, initiating the ATF4 and CHOP pathway. CHOP plays a vital role in ER-stress-induced cardiomyocyte apoptosis in DM. The protein expression levels of PERK, eIf2a, and their phosphorylated form (*p*-PERK and *p*-eIf2a) were investigated. The protein expression levels of *p*-PERK and *p*-eIf2a were significantly increased (*p* < 0.001) in DCM rats compared to control animals. The LCZ696 and valsartan treatment of DCM rats significantly inhibited *p*-PERK (*p* < 0.001) and *p*-eIf2a protein levels (*p* < 0.01) in cardiac cells as compared to the untreated DCM animals. The ATF4 mRNA levels were significantly increased (*p* < 0.001) in the DCM rat group compared to the control rats. The LCZ696 and valsartan treatments significantly reduced the ATF4 mRNA levels (*p* < 0.001 and *p* < 0.05, respectively) compared to the DCM rats. However, the LCZ696 treatment markedly reduced the elevated ATF4 mRNA levels (*p* < 0.01) compared to valsartan-treated animals. CHOP mRNA and protein levels were significantly elevated (*p* < 0.001) in cardiac cells of DCM rats compared to the control group of animals. The LCZ696 and valsartan treatment of DCM rats markedly reduced the CHOP mRNA and protein levels (*p* < 0.001) compared with the untreated DCM animals. Despite this, CHOP mRNA and protein levels in the LCZ696-treated animals were significantly lower (*p* < 0.01) than in the valsartan-treated animals.

### 2.10. Effect of LCZ696 and Valsartan on Histopathological Changes in Heart Tissues

**Figure 10:**
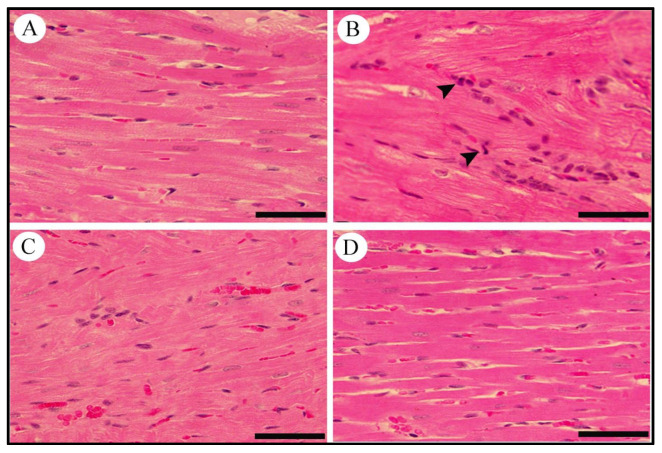
Effects of LCZ696 and valsartan treatments on diabetes-induced histopathological changes in cardiac tissues. (**A**) control, (**B**) diabetic group with inflammatory cells infiltration (arrow head), (**C**) diabetic group treated with valsartan, (**D**) diabetic group treated with LCZ696 (H&E staining, scale bar = 50 µm).

Histological changes were detected in cross-sections of heart tissues from normal and diabetic rats treated with LCZ696 and valsartan. The control group showed the normal appearance of myocardial cells with oval elongated nuclei and homogenous cytoplasm. The heart tissue from diabetic rats shows marked myocardial degeneration associated with focal myocardial disarranging, vessel congestion, and severe intramuscular inflammatory cell filtration. Diabetic rats treated with LCZ696 or valsartan showed a considerable improvement in histopathological abnormalities in most myocardial fibers with a mild degree of myocardial degeneration (Figure 10).

## 3. Discussion

In the present study, we investigated the effects of LCZ696 therapy against DCM in a rat model, and the results obtained were compared with the valsartan treatment as a referral group. LCZ696 markedly inhibited the myocardial inflammation, ER stress, and apoptosis through the RAGE/NF-κB and PERK/CHOP signaling cascades. These activities were found to be substantially higher in the LCZ696 group compared to the valsartan-treated animals. The present results show a significant decrease in cardiac enzyme levels produced by the LCZ696 treatment, and those that were found were more inhibited than those in the valsartan-treated animals. Additionally, a reduction of apoptotic gene markers (BAX/Bcl-2 ratio and caspase-3) was detected in both mRNAs as well as protein expressions in the LZC696 treated group. In addition, inhibition of AGEs formation at the activity level and receptor (RAGE) of the mRNA was induced by LCZ696. Furthermore, an NF-κB reduction was associated with the restoration of elevated proinflammatory cytokines, including the TNF-α, IL-6, and IL-1β that were noticed in LCZ696 treated animals. The LCZ696 treatment restored the induction of ER stress parameters (GRP78, PERK, eIF2a, ATF4, and CHOP), suggesting that LCZ696 reduces DCM in the rats model through its anti-inflammatory, antiapoptotic, and ER stress inhibition effects.

Our findings revealed no significant change in body weight after ten days of diabetic induction in DM rats, but they showed a significantly lower body weight after the 16th day, and that decline continued until the end of the experiment (16 weeks) compared to the control, regular-diet-fed rats. Other studies also showed a reduction in the body weight of animals fed HF with different STZ doses [17,18,19]. The HFD administration followed by the injection of a small dose of STZ stimulated insulin resistance accompanied by the development of hyperglycemia, hyperinsulinemia, and dyslipidemia in the experimental rats; the present results agree with Abdulmalek et al. [20]. In the present study, serum insulin levels were estimated after 16 weeks of diabetic induction by a small dose (30 mg/kg) of STZ. No significant changes were seen in insulin levels when the HFD-STZ and control groups were compared. We believe that when STZ was administered to rats (in the 6th week), β cells were “partially” destroyed; subsequently, there was an increase in the mass by division of surviving cells, increasing the production of insulin. The continuity of a HFD in diabetic rats may imply that the insulin resistance installed by the HF diet was not compensated by additional insulin secretion. Recently, DeMagalhães et al., (2019) also observed similar unchanged serum insulin levels in a HFD-STZ group after 12 weeks [21]. Rankin and Kushner (2009), in their study of the β-cell regeneration ability observed in partially pancreatectomized mice, showed that the β-cell regeneration capacity changes as a function of age [22]. The authors demonstrated that β-cell regeneration was more robust in younger than older (e.g., 12 month old) mice. In the present study, we used adolescent rats. Thus, these facts can justify why the animals in the HFD-STZ group showed unchanged insulin levels at the end of the experiment when compared with the control animals.

Elevations in cardiac enzymes (CK-MB and LDH) are known biomarkers demonstrating cardiovascular disorders that can induce myocardium damage. Several studies reported that CK-MB and LDH are elevated in animals with experimentally induced diabetes [23,24,25]. Similarly, we too observed increased serum CK-MB and LDH levels in diabetic rats compared to the control rats. Consistent with the current findings, it has been demonstrated that valsartan and captopril, angiotensin II converting enzyme inhibitors (ACEi), diminished CK-MB and LDH levels in STZ-induced diabetic rats [26]. However, it was revealed that the inhibition of cardiac enzymes was greater in the LCZ696-treated group compared to the valsartan-treated rats, justifying the positive effect of LCZ696 against diabetes-induced cardiac damage. It was also demonstrated that telmisartan, angiotensin II type 1 receptor blocker and thiorphan, neprilysin inhibitors reduced CK-MB and LDH in STZ-induced cardiomyopathy in the rats model. This reduction of the telmisartan/thiorphan combination was superior to that of telmisartan alone [27].

The activation of multiple ER stress signaling pathways is known as the unfolded protein response (UPR). The UPR is activated when the ER capacity of a folded protein is overloaded due to its cellular demand or the presence of the mutant protein presence to maintain the ER homeostasis and normal cellular function. Furthermore, sustained activation of ER stress signaling triggers the release of the apoptotic pathways as well as cell death [28]. The proinflammatory pathway has been linked to UPR signaling through an elevation of the NF-κB and ROS formation [29]. Induction of the NF-κB by AGEs triggers several signaling cascades via enhancing the production of proinflammatory cytokines, such as the TNF-α, IL-6, and IL1β, which are responsible for the development of cardiac structural and functional alterations in DM [27,30]. We also observed such elevations in TNF-α, IL-6, and IL1β levels in cardiac tissues. These cytokines regulate myocyte survival and death, modulate cardiac contractility, vascular endothelium changes, and recruit other circulating cells of inflammation in injured myocardium. This may lead to further oxidative stress and remodeling. Earlier, it was indicated that diabetes induces myocardial apoptosis in human patients [31] as well as in diabetic animal models [32]. In mouse and rat models of type I diabetes, TNF-α neutralization has been shown to attenuate the DCM, as evidenced by the reduction of intramyocardial inflammation and cardiac fibrosis [33]. The present results also demonstrated that LCZ696 and valsartan reduced levels of the TNF-α, in addition to other proinflammatory cytokines like IL-6 and IL-1β, and results in the preservation of the cardiac structural and functional integrity. Indeed, it has been revealed that at least part of these protective actions are mediated by the inhibition of the NF-κB. These findings are important because we know that increased ROS levels activate the NF-κB and that leads to increased proinflammatory cytokines such as IL-6 and the TNF-α in the human heart [34]. Furthermore, valsartan has been shown to inhibit cardiac TNF-α, IL-6, and IL1β production in a rat model of myocardial ischemia [33]. Consistent with these findings, in our study LCZ696 and valsartan attenuated the TNF-α, IL-6, and IL1β in DCM-induced rats, and that effect was superior in LCZ696-treated rats when compared to the valsartan-treated group. It concludes that the utilization of LCZ696 is a better option for clinical DCM patients.

AGEs accumulation is a well-known parameter in diabetes that mediates the unwanted effect of hyperglycemia. AGEs formation in humans may be implicated in inflammation and apoptosis by the induction of ER stress [35]. Multiple evidences demonstrated a crosstalk between AGEs formation and the activation of ER stress pathways in several cell types [36,37,38]. They found that AGEs mediated UPR singling in various pathogenesis [36]. In our study, AGEs and their receptor (RAGE) accumulation were detected in diabetic animals compared to the control group. Earlier studies demonstrated that aminoguanidine (an inhibitor of AGE formation) treatment in the type-1 diabetic rat model ameliorates the dysfunction of the left ventricle [39]. In the present study, AGEs and RAGE were reduced by the LCZ696 and valsartan treatment. Moreover, the reduction of AGEs and RAGE by LCZ696 was superior to the valsartan treatment. In agreement with our finding, ramipril, an ACEi, was found to reduce AGEs accumulation in STZ-induced diabetic nephropathy [35], and valsartan was also found to markedly reduce AGEs formation in hypertensive type-2 diabetic patients [40].

ER stress is implicated importantly in several pathogeneses, including DCM [41]. GRP78 is one of the ER chaperone proteins involved in inducing protein folding and preventing aggregation. It is considered as an indicator of the ER stress activation process. It has been reported that the systemic effect of AGEs can interfere with UPR function by the induction of GRP78 expression [35]. The GRP78/PERK complexes were found to be in the inactive state under unstressed conditions. At the initiation of UPR, GPR78 relocalized to permit PERK dimerization followed by its autophosphorylation to activate the downstream signaling pathway [41]. It has been reported that GRP78 induced significantly in transgenic non-obese diabetic rats compared to controls. In addition, valsartan has been reported to reduce the cardiac elevation of GRP78 and caspase-3 in diabetic type-1 induced cardiomyopathy rats [42]. Consistent with these results, we observed that LCZ696 and valsartan inhibited the induction of GRP78 in DCM rats, and LCZ696 was superior to valsartan. In a prolonged activation of ER stress, the up-regulation of CHOP is critically involved in ER stress-induced apoptosis. This process regulates three ER stress sensors, including PERK. The initiation of PERK leads to the phosphorylate of eIF2α allowing the transcription of ATF4, which leads to the activation of CHOP [13,43]. Several reports demonstrated the pivotal role of the PERK–CHOP pathway in the induction of the apoptotic cascade in invitro and invivo models [44,45,46]. In addition, Wu et al. (2011) reported that valsartan prevented DCM by inhibiting ER stress in diabetic rats [42]. In our study, LCZ696 reduced the cardiac *p*-PERK and *p*-EIF2α and CHOP induced in diabetic rats. However, the study demonstrates that the cooperation of CHOP and ATF4 is required to induce cell death pathways [47]. In context, we established that valsartan and LZC696 reduced the ATF4 in mRNA level compared to the DCM model.

The role of ER stress in DCM was questionable until 2006, when Li et al. shed light and demonstrated the implication of ER stress in myocardium apoptosis on diabetic rat models [48]. Apoptosis is strongly implicated in the pathogenesis of DCM, which plays a vital role in cardiac dysfunction [49]. In response to hyperglycemia, the induction of inflammation, apoptosis, and ER stress cardiac cells have been reported [50]. Moreover, AGEs induced ER stress resulting in the activation of pro-apoptotic cascades [35]. Several studies reported that apoptotic induction in cardiomyocytes was detected in diabetic animal models [27,32]. The most well-known parameters of pro-apoptosis genes are caspase-3 and BAX and the antiapoptotic gene Bcl-2. The activation of caspase-3 was reported to be associated with the induction of the pro-apoptotic pathway in DCM, and that using a specific caspase-3 inhibitor could attenuate apoptosis [32]. In the present study, the levels of caspase-3 and BAX/Bcl-2 ratio levels were markedly induced in DCM rats compared to the control. In addition, LCZ696 and valsartan reduced the caspase-3 and BAX/Bcl-2 ratio compared to untreated diabetic animals. Consistent with our finding, valsartan diminished the expression level of caspase-3 in diabetic-induced cardiomyopathy in rats [42]. In addition, an elevation of caspase-3 and the BAX/Bcl-2 ratio was detected in diabetic rats, and this elevation was reduced by the LCZ696 treatment. Interestingly, we observed that the reduction of caspase-3 and the ratio of BAX/Bcl2 by LCZ696 was superior to that of valsartan, demonstrating the role of LCZ696 in apoptotic attenuation.

## 4. Materials and Methods

### 4.1. Animals and Ethical Approval

Adolescent male Wistar rats weighing 100–120 g were obtained from the Experimental Animal Care Center, Pharmacy College, King Saud University, where they were maintained and monitored in a specific pathogen-free environment. All experimental procedures, including euthanasia, were conducted in accordance with the National Institute of Health Guide for the Care and Use of Laboratory Animals, Institute for Laboratory Animal Research (NIH Publications No. 80-23; 1996), as well as the ethical, approved Guidelines (SE-20-8) of the Experimental Animal Care Centre, College of Pharmacy, King Saud University (KSU), Riyadh, Kingdom Saudi Arabia (KSA). All animals were allowed to acclimatize in polycarbonate cages inside a well-ventilated room for 7 days prior to experimentation. The animals were maintained under standard laboratory conditions (temperature of 23–24 °C, relative humidity of 60–70%, and 12 h light/dark cycle).

### 4.2. Experimental Design

Control group rats were fed a D12450H control diet (10 kcal% fat, manufactured by Research Diets Inc., NJ, USA) throughout the experiment, whereas the diabetic groups of rats were fed a high-fat diet (HFD) of D12451 (45 kcal% fat, manufactured by Research Diets Inc., NJ, USA). At the end of 6 weeks of feeding, the diabetic groups of rats were injected with a low dose of STZ (30 mg/kg, i.p., Sigma-Aldrich, Germany) freshly prepared in citrate buffer (0.1 M, pH 4.5). Meanwhile, citrate buffer was injected intraperitoneally into the non-diabetic animals as a vehicle dose. Two days after STZ injection, DM was verified by estimating fasting blood glucose levels from the animal’s tail vein using a glucometer (Accu-chek Compact Plus glucose meter system; Roche Diagnostics, Meylan, France). Animals with glucose levels of more than 250 mg/dL were considered to be diabetic. After the induction of diabetes, rats were randomly divided into 4 groups (*n* = 8): (1) normal rats treated with vehicle (Control), (2) DM rats treated with vehicle (DCM), (3) DM rats treated with valsartan (Valsartan), and (4) DM rats treated with LCZ696 (LCZ696). Valsartan, Tabuvan^®^ 80 mg (Tabuk Pharmaceutical Manufacturing Co., Tabuk, KSA) was suspended in 0.5% carboxyl methylcellulose (CMC) and administered 31 mg/kg/day via gastric gavage daily at approximately the same time to normal and diabetic rats. LCZ696 (sacubitril/valsartan, Entresto™ 200 mg; Novartis, Switzerland) was suspended in 0.5% carboxyl methylcellulose (CMC) and administered at a dose of 68 mg/kg/day via gastric gavage approximately daily at the same time to normal and diabetic rats. Treatment was initiated two weeks after diabetic induction and was continued for 8 weeks. Animal body weights were recorded weekly on the same day and at approximately the same time.

During the experiment, blood pressure measurements were performed three times (day 0, before treatment, and at the end of treatment) using tail-cuff instruments and echocardiography, respectively. Subsequently, the rats were anesthetized using ketamine and xylazine mixture (94:14 mg/kg), and blood samples were then collected through cardiac puncture. Blood was centrifuged at 1800× *g* for 10 min at room temperature (Hettich EBA-20; Andreas Hettich GmbH & Co. KG, Tuttlingen, Germany), and the serum was transferred to a new tube and stored at −20 °C. Hearts were immediately dissected, and a small section was fixed in 10% formaldehyde for histopathological analysis. The remaining heart tissue was dipped in liquid nitrogen for 1 min and then stored at −80 °C for further analysis.

### 4.3. Serum Biochemical Assays

Serum glucose, total cholesterol (TC), triglycerides (TG), low-density lipoprotein cholesterol (LDL-c), and high-density lipoprotein cholesterol (HDL-c) levels were assessed by a commercially available kit (Randox Laboratories Ltd., UK and SPI bio, France). Insulin levels were estimated using ELISA system (Merck Millipore, USA). Serum levels of lactate dehydrogenase (LDH) and creatine kinase-MB (CK-MB) were determined using commercially available diagnostic kits (Human, Wiesbaden, Germany). Insulin levels were determined using an ELISA kit (R&D Systems, Inc., USA).

### 4.4. Non-Invasive (Tail-Cuff) Blood Pressure Measurements

Systolic blood pressure (SBP) and diastolic blood pressure (DBP) were measured by the tail-cuff instrument, a non-invasive method for blood pressure measurement, using CODA 20830 (Kent Scientific, USA). Rats were trained for 5 days to acclimatize to the restraint platform and tail-cuff inflation. Animals were kept in the restraint platform at 37 °C for 10 min before the measurement to make the pulsations of the tail artery readable. BP measurements were conducted during the day to avoid the influence of the circadian. The SBP and DBP values were obtained by averaging the readings of 15 measurements. The measurements were taken on day 0, after 8 weeks, and at the end of treatment. BP measurements were conducted during the daytime to avoid the influence of the circadian. SBP and DBP values were calculated from an average of 10–15 readings. Each rat was evaluated at least 3 times per session.

### 4.5. Echocardiography

At the end of the treatment, rats were anesthetized with ketamine/xylazine (94:14 mg/kg) and then immobilized in the supine position, and the chest and abdomen areas were shaved. M-mode recordings of the left ventricle were obtained by placing the liner 12 MHz array transducer system in the parasternal view. The ejection fraction (EF) and fractional shortening (FS) were calculated. For each assessment, data from five consecutive cardiac cycles were obtained.

### 4.6. RNA Extraction and cDNA Synthesis

TRIzol reagent (Invitrogen^®^) was used for cellular RNA isolation according to the manufacturer’s instructions. The quality and quantity of isolated RNA were measured by obtaining the absorbance at 260 nm and maintaining a 260/280 ratio of ~2.0. According to the manufacturer’s instructions, first-strand cDNA was synthesized using the High-Capacity cDNA Reverse Transcription Kit (Applied Biosystems^®^).

### 4.7. Quantification of mRNA Expression Levels by Real-Time Polymerase Chain Reaction (RT-PCR)

The mRNA expression of target genes was quantified using RT-PCR by subjecting the resulting cDNA to PCR amplification using the QuantStudio 6 Flex RT-PCR System (Applied Biosystems^®^) based on SYBR Green Universal Master Mix as previously described [51]. Rat primers for target genes (Table 2) were purchased from Integrated DNA Technologies (IDT, Coralville, IA). The fold change in the level of these genes between all animal groups was corrected by the levels of β-actin. The RT-PCR data were analyzed using the relative gene expression (i.e., ∆∆ Ct) method, as previously described and explained [52] using the following equation: fold change = 2^−∆(∆Ct)^, where ∆Ct = Ct_(target)_ −Ct_(β-actin)_ and ∆(∆Ct) = ∆Ct(treated) −∆Ct(untreated).

### 4.8. Western Blot Analysis

A Western blot experiment was performed using a previously described method [53]. Briefly, 20–25 μg of proteins from each group were diluted in the same amount (1:1) of 2X loading buffer (0.1 M Tris(hydroxymethyl)aminomethane (Tris)-HCl, pH 6.8, 4% sodium dodecyl sulfate (SDS), 1.5% bromophenol blue, 20% glycerol, 5% β-mercaptoethanol) and separated by 10% sodium dodecyl sulfate (SDS)-polyacrylamide gel electrophoresis (PAGE), and then electrophoretically transferred to nitrocellulose membrane. The protein blots were then blocked overnight at 4 °C in blocking solution and then washed several times with TBS-Tween-20 before being incubated overnight at room temperature with primary antibodies against BAX, Bcl-2, cleaved caspase-3, NF-kB, PERK, *p*-PERK, EIf2a, *p*- EIf2a, and CHOP. The primary antibody solution was removed, and the blots were rinsed five times with wash buffer, followed by incubation with peroxidase-conjugated IgG secondary antibodies for 2 h at room temperature. The bands were visualized and then quantified by a C-DiGit^®^ Blot Scanner, LI-COR Biosciences (Lincoln, Nebraska, USA) using the enhanced chemiluminescence method according to the manufacturer’s (Merck Millipore, Billerica, MA, USA) instructions.

### 4.9. Measurement of Advanced Glycation Products Activity and Interleukins in Cardiac Tissues

In heart tissues homogenated in PBS solution, the advanced glycation products level was measured using a Rat AGE (sandwich ELISA) ELISA kit (LifeSpan BioSciences, USA) according to the manufacturer’s instructions. The cardiac interleukin IL-6, IL-1β, and TNF-α levels were estimated using commercially available ELISA kits (R&D Systems, Inc., USA). An ELISA kit (Elabscience Biotechnology, China) was used to determine the serum level of brain natriuretic peptide (BNP) according to the manufacturer’s instructions.

### 4.10. Cardiac Histopathology

Fixed cardiac tissues were dehydrated in a serial ascending dilution of ethanol and xylene and then embedded in paraffin wax. Using an automated microtome (Leica RM 2125 RM, Leica Microsystems, Nussloch, Germany), sections (5–7 µm) were cut and mounted on glass slides. Sections were stained with hematoxylin and eosin (H&E). The slides were examined by an experienced pathologist in a blinded manner using a light microscope (Nikon Eclipse E600) with a high-resolution digital camera.

### 4.11. Statistical Analysis

Data are presented as mean and standard error (mean ± SEM). One-way ANOVA was performed to test the significant differences between groups using Graph-Pad Prism version 8 (GraphPad Software, Inc., La Jolla, CA, USA). Student–Newman–Keuls, a multiple comparison test, was utilized as a post-hoc test. Statistical significance was set at *p* < 0.05.

## 5. Conclusions

In the present study, LCZ696 markedly inhibited myocardial inflammation, the ER stress parameters (GRP78, PERK, eIF2a, ATF4, and CHOP) in mRNA and protein expressions, and apoptosis through the RAGE/NF-κB and PERK/CHOP signaling cascades. Herein, we compared the effects of LCZ696 with valsartan against the DCM and found a higher beneficial effect in the LCZ696 group. Therefore, LCZ696 represents a potential new drug for the treatment of DCM.

## Figures and Tables

**Table 1:** The effect of LCZ696 and valsartan on systolic and diastolic blood pressure in diabetic cardiomyopathy rats.

	Day 0		8th Week		16th Week	
	Systolic	Diastolic	Systolic	Diastolic	Systolic	Diastolic
Control	116.29 ± 8.04	83.07 ± 8.83	114.73 ± 5.88	88.6 ± 5.68	121.71 ± 5.72	79.93 ± 10.47
DCM	115.64 ± 7.72	83.57 ± 7.55	158.57 ± 7.19 *	110.93 ± 7.84 *	175.11 ± 7.03 *	117.21 ± 4.55 *
Valsartan	117.42 ± 3.92	77.33 ± 9.03	153.53 ± 7.32	118.4 ± 8.32	135.67 ± 5.92 #	88 ± 5.89 #
LCZ695	120.93 ± 2.52	80.27 ± 4.32	150.73 ± 7.05	116.8 ± 4.48	134.9 ± 5.22 #	86 ± 4.48 #

Table 1. Data are expressed as mean ± SEM. * DMC was compared to control group; # LCZ696 and valsartan groups were compared to DCM. The level of significance was determined as *p* < 0.05.

**Table 2:** Primers sequences used for real-time PCR reactions.

Gene	5′→3′ Forward Primer	5′→3′ Reverse Primer
**β-actin**	TGT TGC CCTAGA CTT CGA GCA	GCCAGTAGAGGCAGGGATGAT
**Caspase-3**	CGCAGACCTTGTGATATTCCAG	CGTTTCTTCCATCCTTCCAGG
**BAX**	TCCACCAAGAAGCTGAGCGAG	GTCCAGCCCATGATGGTTCT
**Bcl2**	CATGTGTGTGGAGAGCGTCAA	GCCGGTTCAGGTACTCAGTCA
**NF-kB**	AGTCCTGCTCCTTCCAAAAC	CTTCGGTGTAGCCCATTTGT
**RAGE**	TCCTGGTGGGACCGTGAC	GGGTGTGCCATCTTTTATCCA
**CHOP**	CCAGCAGAGGTCACAAGCAC	CGCACTGACCACTCTGTT TC
**ATF-4**	CACTAGGTACCGCCAGAAGAAGA	AATCCGCCCTCTCTTTTAGAG
**GRP78**	TCAGCCCACCGTAACAAT	CAAACTTCTCGGCGTCAT

## Data Availability

The data used to support the findings of this study are included within the article.
